# A Qualitative Endline Evaluation Study of Male Engagement in Promoting Reproductive, Maternal, Newborn, and Child Health Services in Rural Kenya

**DOI:** 10.3389/fpubh.2021.670239

**Published:** 2021-07-08

**Authors:** Adelaide M. Lusambili, Stefania Wisofschi, Constance Shumba, Peter Muriuki, Jerim Obure, Michaela Mantel, Lindsay Mossman, Rachel Pell, Lucy Nyaga, Anthony Ngugi, James Orwa, Stanley Luchters, Kennedy Mulama, Terrance J. Wade, Marleen Temmerman

**Affiliations:** ^1^Department of Population Health, Medical College, Aga Khan University, Nairobi, Kenya; ^2^Centre of Excellence in Women and Child Health, Medical College, Aga Khan University, Nairobi, Kenya; ^3^Aga Khan Foundation, Canada, Ottawa, ON, Canada; ^4^Department of Public Health and Primary Care, International Centre for Reproductive Health, Ghent University, Ghent, Belgium; ^5^Department of Health Sciences, Brock University, St. Catharines, ON, Canada; ^6^Department of Obstetrics and Gynaecology, Medical College, Aga Khan University, Nairobi, Kenya

**Keywords:** male engagement, reproductive health, family planning, Kilifi, Kisii, Kenya FP, Kenya

## Abstract

**Background:** Globally, male involvement in reproductive, maternal, newborn, and child health (RMNCH) is associated with increased benefits for women, their children, and their communities. Between 2016 and 2020, the Aga Khan University implemented the Access to Quality of Care through Extending and Strengthening Health Systems (AQCESS), project funded by the Government of Canada and Aga Khan Foundation Canada (AKFC). A key component of the project was to encourage greater male engagement in RMNCH in rural Kisii and Kilifi, two predominantly patriarchal communities in Kenya, through a wide range of interventions. Toward the end of the project, we conducted a qualitative evaluation to explore how male engagement strategies influenced access to and utilization of RMNCH services. This paper presents the endline evaluative study findings on how male engagement influenced RMNCH in rural Kisii and Kilifi.

**Methods:** The study used complementing qualitative methods in the AQCESS intervention areas. We conducted 10 focus group discussions (FGDs) with 82 community members across four groups including adult women, adult men, adolescent girls, and adolescent boys. We also conducted 11 key informant interviews (KIIs) with facility health managers, and sub-county and county officials who were aware of the AQCESS project.

**Results:** Male engagement activities in Kisii and Kilifi counties were linked to improved knowledge and uptake of family planning (FP), spousal/partner accompaniment to facility care, and defeminization of social and gender roles.

**Conclusion:** This study supports the importance of male involvement in RMNCH in facilitating decisions on women and children's health as well as in improving spousal support for use of FP methods.

## Plain English Summary

Active engagement of fathers and overall spousal participation in reproductive maternal and new child heath (RMNCH) is associated with improved nutrition and improved decisions and actions for the use of antenatal services (ANC), delivery, and post-natal services (PNC). While research from low and middle income countries (LMICs) has linked male engagement projects to improved couple relationships, joint family decision making, increased uptake of family planning (FP), and reduced child mortality; however, male involvement and participation in women's health remains low in Sub-Saharan African (SSA) settings. We conducted a qualitative evaluation to explore how male engagement strategies influenced access to and utilization of RMNCH services, following a 5-year intervention activity that encouraged greater male engagement in RMNCH in rural Kisii and Kilifi, two predominantly patriarchal communities in Kenya.

We conducted 10 focus group discussions (FGDs) with the community members across four groups including adult women, adult men, adolescent girls, and adolescent boys. We also conducted 11 key informant interviews (KIIs) with facility health managers, and sub-county and county officials who were aware of the project activities. We found that male engagement activities in the two sub-counties were linked to improved knowledge and uptake of FP, spousal/partner accompaniment to facility care, and defeminization of social and gender roles. In conclusion, male involvement in RMNCH is key as it is associated with joint decisions on FP and child spacing with improved spousal support for use of FP methods.

## Background

There is growing recognition of men as actors who influence the health of women and children (1, 2). Addressing gender-based inequalities through enhanced women's empowerment demands that men act in a supportive role toward the realization of women's sexual and reproductive health (1). International research demonstrates that active engagement of men and overall partner participation in reproductive, maternal, newborn, and child health (RMNCH) is associated with improved nutrition and improved decisions and actions for the use of ANC, delivery, and PNC (3–13). Contrary to this, men's disengagement has been shown to have deleterious outcomes such as poor child development, poor maternal and child mental health, and low and delayed uptake of ANC services (11, 12).

Similarly, recent but limited research from LMICs such as Bangladesh, Zimbabwe, Mozambique, and Tanzania has linked male engagement projects to improved couple relationships, joint family decision making, increased uptake of FP, and reduced child mortality (13–15). Despite these reported benefits, male involvement, and participation in women's health remains low in SSA settings (14). This lack of involvement and participation is of particular concern in male dominated societies, like Kenya, and threaten women's autonomy and negatively impact health seeking behaviors throughout pregnancy and delivery (16, 17). In such contexts, men determine under what conditions their partners or spouses utilize health and FP resources and leave women unable to make decisions about their own health (2, 18, 19).

While one study conducted in Kenya found that men were reported to be facilitators of positive behaviors by encouraging wives and partners to attend ANC visits and facility-based delivery services (11); our previous study also identified specific barriers to men's participation in RMNCH including gendered cultural norms such as the belief that pregnancy is the sole responsibility of the woman, negative health care worker attitudes toward male engagement and maternity, and community health services infrastructures that are unsupportive of men's participation (19). As empowered women are more likely to attend facility-based reproductive health services, utilize modern FP methods, and experience fewer pregnancy complications, an increased emphasis on male engagement in women's health will assist in preventing reproductive health issues, increasing acceptance of contraceptive methods, and empowering women's decision making (20–22). As such, the promotion of FP initiatives that involve men is key to addressing barriers to men's supportive participation in reproductive and maternal health and has been linked to positive health outcomes for women and children (23–26).

In an attempt to identify and address the barriers to women's access to RMNCH in rural Kenya, in 2015, “Access to Quality of Care through Extending and Strengthening Health Systems (AQCESS)” project, an RMNCH project funded by the Government of Canada and Aga Khan Foundation Canada (AKFC) designed and implemented a range of activities (Table 1) in rural Kisii and Kilifi counties, two predominantly patriarchal communities in Kenya. The evaluation study was conducted after a 4-year intervention with activities that promoted the participation of men in RMNCH. This paper presents findings from the end line evaluation study and focuses specifically on understanding how male engagement strategies influenced RMNCH services.

**Table 1:** AQCESS interventions.

1. Using community health volunteers (CHVs), community health committee (CHCs) members, and community health extension workers (CHEWs), dialogues with men and women were carried out quarterly in each of the 17 Community Health Units (CHU's) on the importance of family planning. Dialogue sessions targeted men and women of all ages. Men, including the heads of households, were recruited across the 17 CHU's and from geographically disconnected villages. Sessions, also known as dialogues, engaged women, men, and adolescents of both sexes. Discussions focused on the barriers to access and use of RMNCH services, the importance of men supporting women in RMNCH, and the need for men and women to work together to improve health outcomes for their families. 2. All CHVs and CHC members were mentored by the AQCESS study team on gender equality in RMNCH with the ultimate aim of involving even those members from hard-to-reach communities. a. Mentorship trainings included information on how CHVs can reach out to men, talk to them, and empower them to support their spouses in RMNCH. b. In addition, the AQCESS project team also conducted quarterly outreach through the health facilities in AQCESS catchment n areas. This outreach had a health education component for health care workers on RMNCH and FP targeting both males and females attending facilities for care. c. Facilities provided edutainment sessions on RMNCH targeting both males and females. 3. Project staff employed models that empowered CHVs/CHCs as change agents, which included training sessions on how to reach the heads of households, Maternal, Newborn, Child and Adolescent Health (MNCAH), gender, and how to deliver and facilitate dialogues. 4. Project staff trained members of health facility committees with respect to challenges in RMNCH and gender responsiveness, specifically on male support, and accompaniment for women attending facility care. 5. Project staff promoted gender empowerment through Ministry of Health forums and through the creation of gender champions with representatives from community leaders, CHVs, CHCs, health facility committees, and county child and gender departments. The role of gender champions was to act as role models and agents of change in challenging negative gender and social norms and supporting adoption of positive practices.

## Methods

### Study Design

A qualitative study.

### Study Setting

The evaluation study was conducted in Kilifi (Kaloleni and Rabai sub-Counties) in southeast Kenya and Kisii (Bomachoge Borabu sub-County) in southwest Kenya where Aga Khan University conducted a RMNCH intervention since 2015. Both Kilifi and Kisii are patriarchal communities and geographically dissimilar rural counties in Kenya. Further details on social-cultural context of Kilifi can be found in our earlier research paper (19).

Table 1 gives a summary of some of the interventions that were led by the AQCESS project team to promote male engagement. The interventions were implemented in these areas over a period of 4 years and were nearing completion when this study was conducted and a detailed description is provided by Lusambili et al. (27). The male engagement strategies were designed to change men's behaviors and increase their support toward women during pregnancy, ANC, delivery, and PNC. The interventions also aimed to enable men to become change agents in addressing socio-cultural and gender inequalities in Kisii and Kilifi counties.

### Methods

The qualitative study assessment employed 10 FGDs with 82 participants and 11 KIIs across the two study sites. Key informants who were aware of the AQCESS project in the past 1 year were purposively sampled by AQCESS project managers for interviews. These included males and females at the county, sub-county, and health facility (HF) levels.

Focus group discussions participants were recruited by AQCESS project field coordinators. Across the two sites, FGDs were conducted separately with female and male CHC members, male and female adult community members, and female and male adolescent community members. To qualify, participants had to have lived in the AQCESS target areas for at least 1 year and have awareness of AQCESS male engagement activities. While efforts were made to secure as great a representation as possible to ensure an unbiased and representative sample, this was balanced necessarily by the need to be familiar with or engaged in the AQCESS project. Adolescents were aged 15–19 and included those who had been involved in AQCESS gender forums.

The qualitative evaluation explored the observed benefits of male engagement, the perceived effectiveness of male engagement strategies in promoting RMNCH, facilitators and barriers to male engagement, and the lessons learned for engaging men in RMNCH. For this paper, we will focus on the evaluation of the benefits of involving men in RMNCH activities.

### Interview Process

Data collection was led by the study Principal Investigator (PI), a qualified qualitative consultant, and a team of experienced research assistants. Actual data collection commenced after securing institutional approval from the Aga Khan University (AKU) Kenya and National Commission for Science Technology and Innovation (NACOSTI/P/19/2768) on December 3, 2019. The research also sought consent from all participants and was granted permission to carry out the research from local HF and CHCs.

Data was collected from January to March 2020 in the local Swahili language. All the study participants were provided with full disclosure and information regarding the purpose of the study, including the benefits and risks. They were also given the opportunity to ask questions before, during, and after the KIIs and FGDs. Focus group discussions for “women adult community members,” “male adult community members,” “male adolescents,” and “female adolescents” were facilitated separately by a qualified facilitator and a note taker of the same gender.

Research assistants were trained on the approved protocol requirements and participants consenting processes prior to performing data collection. Parental/guardian assent was sought for adolescents <18 years of age. All participants provided written consent prior to participating in the study. All KIIs and FGDs were conducted in community spaces deemed convenient and private for interviewees to converse. Focus group discussions consisted of 6–8 people.

### Data Management and Analysis

All audio recordings from the collected interview data were labeled and transferred to a secure laptop at AKU's Monitoring Evaluation and Learning Unit (MERL) and then subsequently deleted from the audio recorders. All reflective field notes and transcripts were stored on a password protected computer and accessibility was limited to the study team. Further, transcripts were anonymized by deleting any references to names and additional identifiers to safeguard participants' confidentiality. Translated and transcribed data was checked by the study PI and study consultant who are Swahili native speakers.

To address reliability and validity, two qualitative researchers read all the transcripts, coded them separately into NVivo 12 Data Analysis Software (QSR International), and proceeded to identify codes, categories, and themes with attention to contradictions across the two sites and diversity of experiences, and to perceptions and attitudes across the different stakeholders. These codes, categories and themes were compared and harmonized. Additionally, the study PI randomly reviewed selected transcripts and compared the final codes, categories, and themes identified by the two preceding coders.

Toward the end of March 2020 and early April 2020, the AQCESS research team held a workshop at both study sites to validate the findings. The workshop, attended by all field staff, local stakeholders, and the research team, confirmed the observed interventions as well as findings on the benefits of involving men in the RMNCH activities.

## Results

Results from our endline evaluative study indicate that the intervention may have shifted behaviors with regard to uptake of FP, spousal support in RMNCH, and improved relationships at the household levels, as summarized below:

I. Improved knowledge and uptake of FP methods
II. Spousal accompaniment for antenatal care and facility-based delivery
III. Shift in traditional social and gender norms

### Improved Knowledge and Self-Reported Uptake of Family Planning

Narratives from study participants suggest that AQCESS male involvement strategies may have improved knowledge levels among husbands and partners relating to the benefits of using FP methods. The sensitization of husbands and wives together on the importance of FP was central to shifting behaviors. Findings revealed that male partners who were targeted during the interventions no longer prohibited their wives from utilizing FP methods. In addition, adolescents benefited from early sex education through sensitizations activities organized by AQCESS staff. Overall, male involvement strategies resulted in improved relationships at the household level. These views were reported by both men and women community members and HF managers.

#### Women Access Family Planning Without Fear

In Kilifi, findings from separate FGDs for male CHCs and adult female community members as well as KIIs with a facility health manager suggest collaborative decisions in uptake of FP.

“*…And even now when my wife is going to the hospital or doing family planning it is easy because the man was the one tough headed but now there are teachings, women can do family planning without fear.”*

***FGD_Male CHC*,** ***Kilifi***

“*… before it was very difficult for a mother to go to the hospital for family planning. Even other women used to plan without informing their husbands; or if you tell your husband he would even beat you, but now they understand, you can collaborate and go together to do family planning.”*

***FGD_ Adult female community*** ***member*,** ***Kilifi***

“*Something else about family planning, back then when you would tell your husband you go for family planning, he would refuse and now, because of the trainings, they accept to accompany us, and we get educated together. And it's going on well…”*

***FGD_ Adult female community member*,** ***Kilifi***

“*… now the women do not get frustrations when seeking for services, like they don't have to hide, they discuss with the partners and they just come openly and say we decided.”*

***KII_Health Facility Manager, Kilifi***

In Kisii, however, similar but limited findings were reported from a female adolescent FGD participant.

“*It's good because even men nowadays this issue of family planning they don't leave it to women alone. You find now they discuss how to get few children and they plan so they don't leave it alone to women to do family planning.”*

***FGD_ Adolescent female community member, Kisii***

#### Adolescent Sex Education

Similarly, data also suggests that men's support for and engagement in FP training sessions may have improved adolescent men's knowledge of protective sex behaviors. In Kilifi, adolescent girls also indicated that they were educated during forums organized by AQCESS on youth's dangers and risks of HIV contraction and methods for early pregnancy prevention through the use of contraceptives.

In Kisii, for example, findings from the male adolescent FGDs revealed that young boys may have been taught how to use protection during sex by other men engaged in AQCESS activities.

“*… as we have been involved, they advise us on how we should be staying and when any other youth gets to hear about something, let me say about adult matters, and the young person wants to explore, to know how they happens, but as we attend in the baraza [local meetings] we are advised to wait until we are mature enough.”*

***FGD_Adolescent male community member*,** ***Kisii***

“*…taught how I can protect myself when in a relationship with a lady and you are told to wait until you get mature, you get advised by the doctors then agree on what to do.”*

***FGD_Adolescent male community member, Kisii***

Similar findings were reported in Kilifi from adult male community members and adolescent female community members. In the following quote for instance, an adult male community member mentioned how they now take initiative to impart knowledge on protected sex to the youths.

“*… our youths used to be people that were walking idly but since they were made aware of this issue [early sex] they got directions. Because every time they got training there were lessons taught to those especially about protective sexual practices if they had to have sex. After being taught we go ahead and teach our youths that whenever they get a partner they should not easily trust the partner but if they happen to be in a hurry they should always use protection before involving in sexual intercourse.”*

***FGD_Adult male community member*,** ***Kilifi***

“*A while ago, you know some time back there was HIV/AIDS, long ago there was no education about it, we had knowledge about it, but we only knew that it is there and that we can get HIV/AIDS through sex and we didn't know any other ways. But now there are professionals that have been chosen who tell the youth of all the other ways you can contract HIV/AIDS …”*

***FGD_ Adolescent female community member, Kilifi***

Additionally, female adolescents further reported how the ACQCESS project may have impacted them to take up contraception to prevent early pregnancies.

“*Back then you would find yourself at home idle you don't have work. So, you decide to find someone to marry you. But since this program came, AQCESS, it has taught us to avoid these early pregnancies, through things like family planning, pills and injections that you can take in order to avoid it.”*

***FGD_Adolescent female community member, Kilifi***

#### Joint Decision Making on Family Planning Linked to Improved Relationships

Study participants reported that the use of FP had improved relationships at the family level as husbands and wives have become more united in joint decision-making regarding child spacing. Men's support in child care improved. Findings from Kilifi and largely from both adult female and male community members give details about the shift in the behaviors of men toward joint decision-making regarding FP. In the following three quotes, adult female community members for this study explain their frustrations before their husbands were educated about FP compared to after the intervention, and the changes they have seen since attending trainings through AQCESS activities.

“*First, I say thank you to AQCESS, I once gave birth to twins and whenever I asked my husband to assist me with babies, he used to refuse, but after attending these educative sessions, he helps me carry them to the facility, they are healthy because of the knowledge we gathered and I say thank you to this project.”*

***FGD_Adult female community member*,** ***Kilifi***

“*As for me, what I have learnt is in involving men, its being close to your husband, the kind of love that you share, then if you would start considering issues to do with family planning he will understand you, and planning on how to give birth to children, kids will be educated well, you can build a home, would be having time to buying assets to put in your home, through doing family planning.”*

***FGD_Adult female community member*,** ***Kilifi***

“*Regarding the changes at the moment, its easier at the facility to use those services because back then, men would completely refuse but now I bring my husband, we are counseled well together and we both understand even family planning is explained with him around until I finish, that's why we have improved because back then we were so behind in family planning.”*

***FGD_Adult female community member, Kilifi***

In particular, men pointed out how joint family decision has brought happiness in the home.

“*…as for me, to add is that, emphasizing on the information about family planning, it has brought happiness in homes because it has a created a good relationship between the father, mother and that child. … happiness can be found in those homes, people are staying well and in an organized manner.”*

***FGD_Adult male community member*,** ***Kilifi***

“*So right now, we are on the same path, there is peace in homes, and marriages are being mended, things are being done without opposition. To add on that, most marriages used to break because the man did not know if the woman had done family planning, because the woman did not involve him. So, he will be struggling to get his wife pregnant but in vain because the woman did not tell him, which later brings misunderstanding between them. As my friend had said men have been involved and the field doctors have taught.”*

***FGD__Adult male community member, Kilifi***

In the same vein, scant findings from Kisii further report on the impact of AQCESS in improving their knowledge about FP and child spacing.

“*…I would like to say thank you, because of the doctors from AQCESS, because we have been involved in family planning. As men, we never knew that once a woman delivered needed space, rather we knew that we were to continue getting another child. But we have now improved and we have been trained that after a mother has delivered, she needs to be given more time as you have agreed. You need to discuss together if it is two to three years to enable the kid to grow and also to get enough money to feed and educate the baby as well get sufficient time to advance in life.”*

***FGD_Male CHC, Kisii***

The narratives from participants indicate that the male involvement strategies to some extent may have increased women's agency in accessing FP services. This was largely due to the creation of a supportive and enabling environment resulting in a situation where men and women were able to make joint decisions on FP and child spacing, and were ultimately thriving in joyful and fulfilling relationships. Previous clandestine use of modern FP methods due to fear of domestic violence against women was reported to have been reduced as a result of improved knowledge and support from male partners. The quotes from male adolescents highlight that men's support for, and engagement in FP training sessions increased their knowledge on protective sex behaviors identifying potential generational differences.

### Spousal Accompaniment for Antenatal Care, and Facility-Based Delivery

Our findings across the two sites revealed that male involvement strategies resulted in behavior change and positive practices toward RMNCH as men became more responsible and supportive partners in RMNCH issues. Participants from the study sites reported observed changes in men's behavior, specifically they were seen accompanying their spouses to RMNCH services. In Kilifi, HF managers noted that they had witnessed an increase in spousal accompaniment in the past 3 years.

“*When AQCESS came nowadays we can see at least, let's say in a number of ten, five usually come with their male partners for the services.”*

***KII_Health facility manager*,** ***Kilifi***

“*…this is through the male accompanying their partners and even allowing women to come for this service …and also for the ante-natal clinics.”*

***KII_Health Facility Manager, Kilifi***

Similar sentiments were reported in Kisii among both female and male CHCs members. Participants reported that, prior to the intervention, spousal accompaniment was limited as men were afraid to accompany their wives to attend facility care. However, this improved following the AQCESS interventions.

“*Men have become loving and caring, when he sees that his wife has conceived, he treats her well and when it reaches the labor time, he takes her to the hospital.”*

***FGD_Female CHC*,** ***Kisii***

“*To add on this, previously before AQCESS came in, like most men would not bring their wives to the clinic but nowadays, if you just stand outside a facility, you will find a husband and a wife together in the facility…”*

***FGD_Male CHC*,** ***Kisii***

“*What I can add, nowadays if a woman is expectant, a man is not afraid to take her to the hospital to deliver.”*

***FGD_Male CHC, Kisii***

In Kisii, men were also observed to assist in taking their children to the hospital and were now mindful of the health of their wives and children.

“*Even when taking their children to the hospital they take them both [man and woman], so the father is concerned about the children.”*

***FGD_Adolescent female community member*,** ***Kisii***

“*…men have started to understand the care and health promotions of the mother, children. Now there is this attitude that it is the mother who is supposed to be responsible to ensure good health of the baby, they have reached a point where they have known that even the husband can bring the baby to clinic and they have buried those bad attitudes of saying that clinic is only for the mothers.”*

***FGD_Female CHC, Kisii***

Participants reported behavior change in relation to the adoption of positive RMNCH practices. These mainly manifested in the form of spousal accompaniment for antenatal care and facility-based delivery, which was not previously the case due to lack of knowledge and negative gender norms and sociocultural attitudes. Health facility managers, men, women, and adolescents all echoed this change indicating that many male partners exhibited improved attitudes and behaviors toward their pregnant wives and young children. The men were described as being caring, demonstrating concern about their children's well-being and health, and exemplifying support for their wives by accompanying them to health facilities for antenatal care visits and delivery.

### Shift in Traditional Social and Gender Norms

#### Men Now Perform Traditionally Feminized Roles

Findings chiefly from Kisii showed that male engagement in RMNCH promotion shifted men's views and practices in relation to traditionally feminized roles as men began helping their wives with household chores.

“*Most of the time if we are pregnant they help us in doing house chores… Now they don't leave the work to us. They help out they can do the laundry. If you have been told bed rest, he is the one does that and things like that.”*

***FGD_Adult female community*** ***member***, ***Kisii***

“*I would like to add that since they started to be involved, they fetch water and bring it to the house they also help to carry the baby when he's crying and you're doing something else. When it reaches time to cook if you are cooking ugali [cornmeal] and the child is asleep they help in cutting up the kales.”*

***FGD_Adult female community member*,** ***Kisii***

“*Mostly, from the question you asked, we as men, when my wife is expectant, I can help her to do some house chores like cooking. But previously, that was the work of a wife. She could even cook within a day of delivery. But nowadays, through the public barazas, we as men have been able to be enlightened.”*

***FGD_Adult male community member, Kisii***

Similar but limited findings were reported in Kilifi.

“*But it's like we were oppressing the women back then when we left to them all those duties, but now there are changes, where the husband also helps the women in like cooking and also helping the children in bathing them, washing their clothes, those are the changes that are existing now and they couldn't be there, it's through AQCESS project and gender.”*

***FGD_Adult male community member, Kilifi***

#### Girls Go to School

In addition, involving men and training them on gender equality allowed men in Kilifi to reflect on discriminatory traditional customs, guided them to denounce such practices and move toward recognizing the importance of educating girls and including them in the family inheritance.

“*…When AQCESS was teaching us about gender, you know here at home we had discrimination, my child couldn't own my wealth because we would say she would go to another homestead but we saw it not right that the child is yours and… (Coughs). You fail to give her inheritance.”*

***FGD_Male CHC*,** ***Kilifi***

“*We used to oppress our ladies by not taking them to school, through AQCESS in the issue of gender we are now taking our girl children to school, because back then we used to say a girl child is to grow then get married and so was it.”*

***FGD_Male CHC, Kilifi***

Based on these narratives, male involvement was the key ingredient that led to the positive changes realized. Participants from both Kisii and Kilifi acknowledged this approach in the light of the positive returns it yielded.

“*It is because majority of the Kisii man are the decision makers, so if the decision maker is not involved in the health care of his family, because many women are not the decision makers in this area, then we may not have the desired change.”*

***KII_County official*,** ***Kisii***

“*Yes, when they involved men was that, as you have put, it was that, because automatically the catch and community of this area, we call them the “Mwenye factor”, and if you didn't involve their men then you could not see their women.”*

***KII_Sub-county official*,** ***Kilifi***

“*In the past it was that whatever a man said was final but these days they agree together, the father gives his opinions and the mother gives her own opinions then they get the solution.”*

***FGD_Adolescents female community member, Kisii***

In Kisii, customary laws that led girls to suffer by being circumcised seemed to have shifted, improving girls' health and opportunities.

“*When we explained to them that it is not good to circumcise a girl child because you will cause trauma to her life because there are so many diseases these days and you might not know how one contracts a disease of any kind. So when we gave them those reasons they were all happy and said that they no longer follow the analog way, they follow the digital way, we will no longer follow our past beliefs we will do according to the health because we have to follow the health instructions and if we follow the analog things we will cause injury to ourselves.”*

***FGD_Female CHC, Kisii***

The narratives demonstrated a positive shift in social and gender norms in the intervention communities. Men were reported to be supporting their pregnant wives with household chores, which were previously considered part of women's roles and responsibilities. The project also helped to improve men's understanding of discriminatory practices against the girl child, and allowed them to recognize the importance of according them educational and inheritance rights, in contrast to the past when they believed that a girl child was raised to be married off and would not provide a good return on (educational) investment. The mind-set shift due to the community level dialogue sessions enabled these communities to respect the rights of women and girls and make some commendable strides toward gender equality.

## Discussion

This paper presents findings regarding the influence of male engagement interventions on RMNCH in rural Kilifi and Kisii counties. Our results show that the AQCESS male engagement interventions improved men's knowledge regarding the benefit of FP, which allowed partners to access FP without fear. Interventions also improved adolescents' sexual education, spousal joint decision making and support, and de-feminization of social and gender roles. The findings also show that men's involvement in RMNCH, increases their ability to influence adolescents to adopt protective and mutually beneficial sexual practices for them and their partners.

The reported improvement in spousal support is a positive finding demonstrating that the intervention influenced spousal support for RMNCH in a positive way. Our findings, regarding increased partner support, improved joint decision making on FP and boosted happiness and peace at home, and are consistent with other studies in both LMICs and high income countries (HICs) (3–6, 11, 14). These findings exemplify that it is possible, through dedicated and contextualized interventions, to change gender and social norms related to male involvement in RMNCH. In addition, the progress on access to education for girls illustrated the need for more targeted strategies from the Kilifi county government that may help to sustain these benefits. Encouraging more girls to continue to go to school could help address the high rates of teenage marriages and pregnancies plaguing the region (28).

There are several implications for policy initiatives based on our findings. First, there must be strategies and guidelines to specifically address increased male involvement in the delivery of RMNCH programs. Secondly, metrics, evaluation frameworks, and best practices should be developed and collected to measure how well programs are doing in terms of ensuring that men are involved in RMNCH programs including the use of key, standardized indicators of male involvement. This will ensure that these practices can be adapted and scaled across a wide range of socio-cultural and geographic settings and particularly in LMICs. Third, to encourage greater male participation, attention should be paid to the infrastructure of RMNCH facilities similar to that addressing disrespectful maternity care (19) such as staff training, addressing gender bias among staff, and ensuring that men feel welcome through things such as partner-friendly promotional materials. Most importantly, emphasis should be placed on building upon the set of positive strategies through which programs can support male involvement as demonstrated in this paper. This would ensure that all stakeholders in the implementation sites design and embed complementary strategies in their future policies that can assist in sustaining this progress at the household, community, and national levels.

Consistent with previous work examining male participation in RMNCH that finds positive effects on child health and mortality, maternal health, and improved couple relationships (3–15) our findings demonstrate the potential for further research to deepen our understanding of the processes that can sustain the benefits of engaging men in RMNCH. For instance, future longitudinal qualitative and quantitative follow-up studies across the two sites could help us to understand the extent to which such interventions are sustained after the completion of the project. Research on the key influences of men's lack of support for RMNCH in different age groups could help policy makers identify gaps as well as develop targeted tools and strategies to address and eliminate these barriers. Lastly, a quantitative study with targeted questions on male engagement and epidemiological outcomes related to RMNCH has the potential to fill in gaps not addressed by qualitative research and assess whether these targeted interventions may permeate the larger community.

## Strengths and Limitations

This study and its results are supported by several strengths. First, it used both FGDs and key-informant interviews as data collection methods and had a diversity of participants (CHCs, HF mangers, gender representatives, and both adult and adolescent male and female community members) enabling the researchers to have a deeper understanding of male-involvement in the intervention catchment area. Second, while work has been done in other LMIC countries including Bangladesh, Zimbabwe, Mozambique, and Tanzania (13–15), this study examined one of a few interventions addressing the challenge of increasing male involvement in RMNCH projects that has been evaluated scientifically in rural Kenya which is traditionally male-dominated (2, 16, 17). Lastly, the evaluation was conducted after 4 years of intervention, which was ample time for participants to realize its benefits.

The principal limitation of the study is that it includes only participants who are familiar or involved with the interventions and AQCESS field staff, which may have introduced bias in their narratives, efforts were made to ensure the community focus group participants were representative of their respective communities, However, being insiders, as participants they may not have critically reflected on the benefits or/and they may have been prejudiced by their involvement with the AQCESS project staff, who also recruited them in the study. Second, as a qualitative approach, we did not have control over our interviewees' narratives, and even though the project staff recruited interviewees who were conversant with AQCESS projects, there are many other development programs in Kilifi and Kisii including projects geared toward reproductive health that may potentially flow over into these results. To minimize this potential, it was necessary to recruit persons familiar with AQCESS at the risk of recruitment bias identified above. The extent to which all the benefits reported by the participants are attributable solely to AQCESS-specific interventions could be further explored in non-AQCESS areas.

## Conclusions

This study has shown that male involvement in RMNCH is critical in facilitating decisions on women and children's health and in improving spousal support for the use of FP methods. This indicates the importance of implementing male-involvement focused interventions to enhance RMNCH outcomes in settings with deeply entrenched patriarchal social and gender norms. Similar programs that aim to increase male involvement in RMNCH should systematically examine how individual and community level factors influence male involvement within specific contexts in an effort to further program and policy development.

## Data Availability Statement

The datasets presented in this article are not readily available due to the confidential nature of the data. Requests to access the datasets should be directed to Dr. Adelaide Lusambili and Prof. Marleen Temmerman adelaide.lusambili@aku.edu and marleen.temmerman@aku.edu.

## Ethics Statement

The studies involving human participants were reviewed and approved by National Commission for Science, Technology, and Innovation (NACOSTI) (NACOSTI/P/19/2768) on 03/December 2019. Consent was sought from all the participants and in the communities in which this study was conducted. Written informed consent to participate in this study was provided by the participants legal parents or next of kin.

## Author Contributions

AL: conceptualization, data collection, data analysis, supervision, visualization, writing, and validation. SW: formal analysis, writing, visualization, and validation. CS: interpretation, writing, visualization, and validation. TJW: manuscript review, interpretation visualization and validation. MT: overall PI of the AQCESS implementation and the MERL Unit. All authors contributed to the article and approved the submitted version.

## Conflict of Interest

The authors declare that the research was conducted in the absence of any commercial or financial relationships that could be construed as a potential conflict of interest.

## Abbreviations

AKFC: Aga Khan Foundation Canada
AKU: Aga Khan University
ANC: antenatal care
AQCESS: access to quality care through extending and strengthening health systems
CHC: community health committee
CHEW: community health extension workers
CHU: community health unit
CHV: community health volunteer
FGD: focus group discussion
FP: family planning
HF: health facility
HIC: high-income countries
KII: key informant interview
LMIC: low- to middle-income countries
MERL: monitoring and evaluation and research learning
MNCAH, maternal, newborn: and child health
NACOSTI, national commission for science, technology: and innovation
PI: principal investigator
PNC: post-natal care
RMNCH, reproductive, maternal, newborn, child: and adolescent health
SSA: sub-Saharan Africa.
